# Genome-wide association studies identify new melanoma susceptibility loci

**DOI:** 10.1186/1897-4287-10-S3-A5

**Published:** 2012-04-20

**Authors:** Tadeusz Dębniak

**Affiliations:** 1 Pomeranian Medical University, Clinic of Oncology, Szczecin, Poland

## 

We report results of the genome-wide association studies for melanoma that were conducted by the GenoMEL Consortium. In the first study 2981 individuals with melanoma and 8408 study-specific control individuals of European ancestry were analysed, in the second experiment 2168 Australian individuals with melanoma and 4387 control individuals were genotyped for 317,000 or 610,000 single-nucleotide polymorphisms (SNPs). Three of the previously reported melanoma susceptibility loci (MC1R, ASIP and CDKN2A) reached genome-wide significance in both studies. Three new loci were found to be associated with melanoma risk in European GWAS: rs1801516 in ATM, rs45430 in MX2, rs13016963 in CASP8. A fourth locus near CCND1 remains of potential interest. An Australian GWAS identified a new susceptibility locus at 1q21.3 (rs 7412746) and potentially 1q42.12 (rs3219090). Further studies will be required to determine which gene or genes at the reported loci mediate melanoma risk.

